# Double Negativity in 3D Space Coiling Metamaterials

**DOI:** 10.1038/srep33683

**Published:** 2016-09-21

**Authors:** Santosh K. Maurya, Abhishek Pandey, Shobha Shukla, Sumit Saxena

**Affiliations:** 1 Nanostructures Engineering and Modeling Laboratory, Department of Metallurgical Engineering and Materials Science, Indian Institute of Technology Bombay, Mumbai, MH, 400076, India

## Abstract

Metamaterials displaying negative refractive index has remarkable potential to facilitate the manipulation of incident waves for wide variety of applications such as cloaking, superlensing and the like. Space-coiling approach is a recently explored technique to achieve extreme properties. The space coiling phenomena cause less energy absorption as compared to local resonating phenomena for obtaining extreme parameters. Here we show extreme properties in doubly negative 3D space coiling acoustic metamaterials. Frequency dispersive spectrum of extreme constitutive parameters has been calculated for 2D maze and 3D space coiling labyrinthine structure. This is in good agreement to the calculated acoustic band dispersion.

Manipulation of waves by artificially engineered structures has led to promising applications such as cloaking, sub-wavelength focusing^1^,^2^,^3^,^4^,^5^, surface wave manipulation, extraordinary transmission^6^,^7^,^8^,^9^ to mention a few. This concept has been extended to acoustic waves. Akin to electromagnetic response in optical metamaterials, acoustic metamaterials are designed by demanding effective mass density and bulk modulus to be negative. Acoustic metamaterials can be categorized into inertial acoustic metamaterials^10^, intrinsic acoustic metamaterials and phononic^11^ crystals. Acoustic metamaterials demonstrate extreme properties by creating local resonances using dampers; single acoustic functional scatterers etc. in a matrix. Space coiling is a recently discovered phenomenon and an alternative approach for achieving extreme acoustic parameters. It is understood that these do not utilize the concept of any local resonances. Recent advances have shown that double negativity can be achieved by coiling up space in 2D^12^. Since the enhancement in the path length and phase delay in the propagating wave is correlated to the coiled up space inside the structure, the effective medium description of structure is valid. This mimics a very high refractive index material which could be used as an effective medium at low frequency for getting extreme acoustic parameters and wavefront modulation^13^. These are also reported to show Mie resonances^14^. It therefore seems possible to design 3D acoustic metamaterial space coil structure showing band folding at desired frequency region. Recent investigation of 3D labyrinthine structure has shown systematic attenuation in transmitted acoustic waves^15^. Here we report extreme acoustic properties in these 3D space coiling labyrinthine structures. Our finite element modeling calculations show that these space coiling structures demonstrate doubly negative properties (effective mass density and bulk modulus) in the low frequency regime. These results are in good agreement with the acoustic band structure calculations.

The space coiling structures allow the incident wave to propagate only through the small orifices into the metamaterial structure. The transmitted wave in space coiling acoustic metamaterial structure for different frequencies gets attenuated. This attenuation in amplitude of the incident interface can be attributed to the acoustic impedance which determines the amplitude of total reflected acoustic pressure of the wave impinging normally on material surface. Since acoustic wave has a scalar field nature, it is therefore possible to coil up wave using 3D perforation inside the structure. The sub-wavelength dimensions of the 3D metamaterial unit qualify them to be considered as an effective medium for wave propagation. This sub wavelength nature of maze in 2D metamaterial structure and impedance mismatch between material and medium has been understood to lead to formation of dispersion characteristics. The use of effective medium approach can be used for extracting extreme properties. The *S*-parameter retrieval methods have been used for characterizing artificially structured metamaterials. These structures by nature are generally inhomogeneous. This concept was initially introduced by Smith *et al*. for electromagnetic metamaterials^16^, and has been subsequently modified by for the acoustic waves. In this approach, the effective refractive index *n* and impedance *Z* are obtained from the complex reflection and transmission coefficients (or *S* parameters) for a plane wave normally incident on the slab. Following this, the effective mass density and speed of sound is calculated. This technique can be used for obtaining the effective properties both using experiments and simulations. The impedance (*Z*) and the refractive index (n) can be expressed in terms of the reflection (*S*_*11*_) and transmission (*S*_*21*_) coefficients using Equation no. 1 and Equation no. 2 respectively
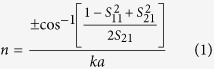
and
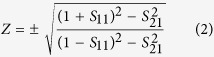
Here, k is the wave number and a is the unit cell dimension. The effective density (ρ) and bulk modulus (B) relative to the background fluid can be obtained from n and Z using the Equation no. 3 and Equation no. 4 respectively.
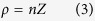
and
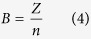
In order to calculate the extreme acoustic parameters, the 2D maze and 3D labyrinthine structure can be represented by effective equivalent structure as shown in Fig. 1(a,b) respectively. The complex reflection and transmission coefficient were obtained by inserting equivalent structure in a rectangular waveguide. The blue color in equivalent cell diagrams in Fig. 1 represents the effective high refractive index material and other regions represent solid aluminum for simulations.

The calculated response of n, Z, ρ and 1/B for 2D maze is shown in Fig. 2(a,b). These responses are in good agreement with the previously reported experimental and theoretical data^17^. Similar approach using *S*-parameter retrieval method described above was utilized to calculate these parameters for 3D labyrinthine space coiling structures and is shown in Fig. 2(c,d). The plot of ρ and 1/B in Fig. 2(a,c) shows frequency bands where both the parameters are simultaneously negative for both 2D maze (~1 KHz–1.7 KHz and 2 KHz–3.2 KHz) and 3D labyrinthine (~1 KHz–1.4 KHz; ~2 KHz–2.4 KHz; and ~3 KHz–3.5 KHz) space coiling structures. Since n and Z are related to ρ and B, this results in observation of negative refractive index in the above mentioned frequency ranges for both 2D and 3D space coiling structures. These extreme properties can be understood in terms of band folding observed in the acoustic band structure which arises due to the impedance mismatch of the metamaterials with ambience and can be used for bandgap engineering. The dispersion relation has been derived for 2D maze using Floquet Bloch theory as cos *ϕ*_*OA*_ + cos *ϕ*_*OB*_ = 2 cos *n*_1_ *ka*. Here *ϕ*_*OA*_ and *ϕ*_*OB*_ represent the phase change for the Bloch wave in the direction OA and OB as shown in Fig. 1, *n*_1_ is the effective refractive index of the medium, *k* is the wave number and *a* is a dimensional parameter of the metamaterials unit cell^12^.

Similarly dispersion relation can be derived for 3D labyrinthine structures in terms of *ϕ'*_*OA*'_ and *ϕ'*_*OB*__'_, the phase change for the Bloch wave in the directions *O*'*A*' and *O'B'*. The acoustic band dispersion for both 2D and 3D space coiling structures has been plotted in Fig. 3. The 2D and 3D band structure share similar characteristics in terms of slopes in the ΓM and ΓX directions for 2D and ΓP and ΓN directions for 3D case. This indicates the occurrence of band folding in both these structures. For odd multiples of π in the expression for band dispersion obtained using Floquet Bloch theory the total phase change is negative, this results in negative value of refractive index for a given band of frequencies. This is consistent with the results obtained from *S*-parameter retrieval method for both 2D and 3D space coil structure in Fig. 2(b,d). Since the 3D labyrinthine structures offers more effective coiling of space resulting in higher effective refractive index of the sound propagating medium, for a constant value of cos(*nka*) the value of *k* will automatically be reduced. This results in blue shift in frequency response of the 3D structures as seen in Fig. 2(c) when compared to 2D maze structures.

The effect of metamaterial structure on acoustic wave propagation was further investigated theoretically and experimentally, the transmitted acoustic pressure was plotted along the direction of wave propagation within the waveguide structure for various frequencies as shown in Fig. 4(b). Attenuation in propagation of wave is observed both experimentally and computationally. Since the acoustic wave is allowed to propagate only through the orifices. Attenuation in amplitude of the incident interface can be attributed to the acoustic impedance which determines the amplitude of total reflected acoustic pressure of the wave impinging normally on material surface. Acoustic band structure for 3D Labyrinthine structure in Fig. 3(d) shows band folding at very low frequencies. Symmetry in folding of acoustic bands is reflected in attenuation of the total acoustic pressure about the band gap frequencies. Enhancement of total transmitted pressure in the experimental data ~3.5 KHz is attributed to the increased sensitivity of the microphone (inset Fig. (4b)).

To conclude, here we report extreme acoustic properties in 3D labyrinthine space coiling structures. Our calculations for 2D space coiling structures are in agreement with experimental and computational data, thereby validating our computational methodology. Our calculations using *S*-parameter retrieval method shows that these structures show negative values for ρ and B simultaneously in a given band of frequencies. These results are in good agreement with the acoustic dispersion band obtained using Floquet Bloch theorem. Thus 3D labyrinthine space coiling structures are doubly negative.

## Methods

Finite element modeling was used to simulate the *S*-parameters for obtaining the acoustic properties. The material parameters used were isotropic. The equivalent structure was made of Aluminum with channels containing high refractive index material. The density of Aluminum was taken to be 2700 Kg/m^3^ and speed of sound was taken to be 6420 m/s. The high refractive index material was modeled with material of density 84.672 Kg/m^3^ and speed of sound in the medium was defined to be 42.875 m/s. Inhomogeneous Helmholtz equation was used as acoustic wave equation. The meshing used contained 27348 tetrahedral elements, 6215 triangular elements, 978 edge elements and 146 vertex elements. The minimum and maximum element size taken was 0.072 and 0.576 units. The simulations were performed in frequency range from 1 KHz to 4 KHz.

**Figure 4:**
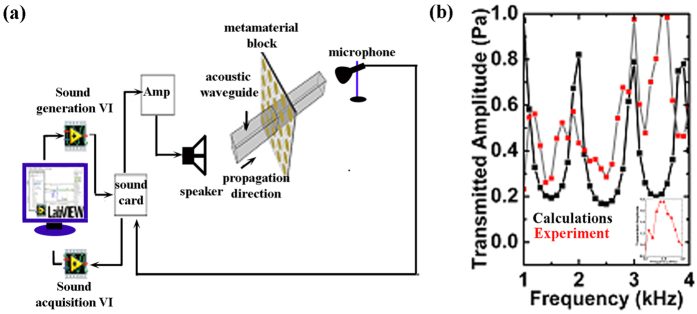
(**a**) Experimental setup for measuring transmission. (**b**) Transmitted data through single layer of 3D metamaterial structure. Red squares represent the experimental data while the black squares represent the calculated data. The inset shows the frequency response of the microphone in the frequency region 3 KHz to 4 KHz.

3D unit cells were fabricated by 3D printing of ABS. These structures are such that acoustic wave is able to propagate in all three directions with same extended path length. Transmission measurements were performed by stacking several 3D unit cells together inside a waveguide with an acoustic wave source at one end and a detector at the other. The acoustic signals of specified frequencies (1–4 KHz) were generated using virtual instrument via a commercial 5 Ohm speaker attached to the soundcard of the computer as shown in Fig. 4(a). This speaker was powered by an amplifier to amplify the generated sound waves using an amplifier. Sound signal was acquired by a microphone attached to onboard soundcard. Response of metamaterial structure containing 5 × 3 × 1 (L×W×H) blocks in the frequency range of 1–4 kHz is reported.

## Additional Information

**How to cite this article**: Maurya, S. K. *et al*. Double Negativity in 3D Space Coiling Metamaterials. *Sci. Rep.* **6**, 33683; doi: 10.1038/srep33683 (2016).

## Figures and Tables

**Figure 1:**
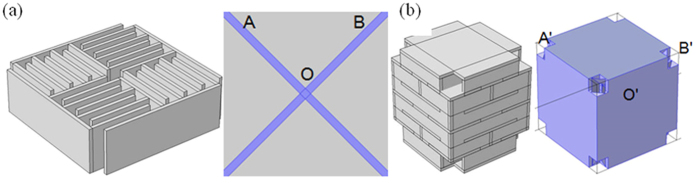
The space coiling cell and equivalent cell for (**a**) 2D maze and (**b**) 3D space coiling labyrinthine acoustic metamaterial structures. O and O’ represents the center of the cell. The blue region represents region of high refractive index.

**Figure 2:**
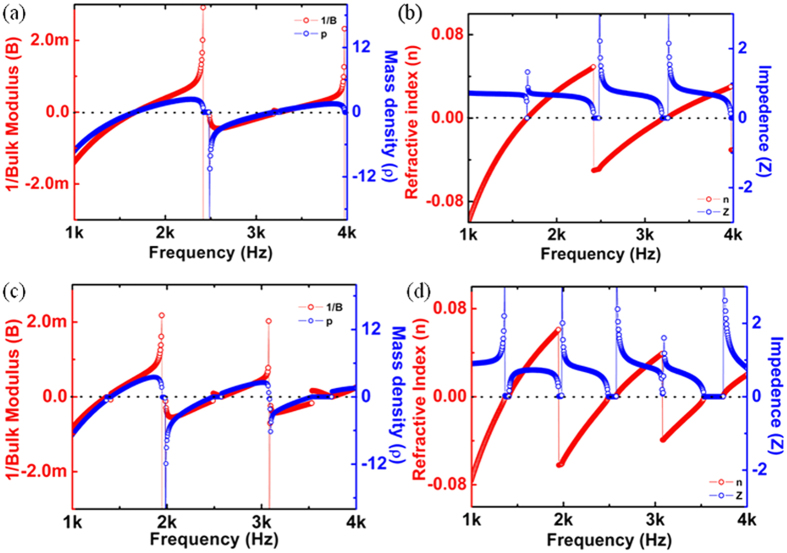
Calculated frequency response of 2D maze for (**a**) 1/B and ρ, (**b**) n and Z. The frequency response of 3D labrythine structure for (**c**) 1/B and ρ, (**d**) n and Z.

**Figure 3:**
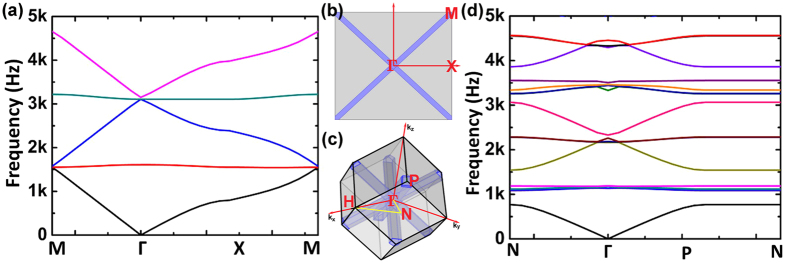
(**a**) Acoustic Band diagram for 2D maze structure. The high symmetry directions are marked on the reciprocal structure for 2D maze in (**b**) and for 3D labyrinthine structure in (**c**). (**d**) Acoustic dispersion for 3D Labyrinthine space coiling metamaterials structures.
